# Fungi Sensitization in Spain: Importance of the *Alternaria alternata* Species and Its Major Allergen Alt a 1 in the Allergenicity

**DOI:** 10.3390/jof7080631

**Published:** 2021-08-03

**Authors:** Verónica P. López Couso, Miguel Tortajada-Girbés, David Rodriguez Gil, Jorge Martínez Quesada, Ricardo Palacios Pelaez

**Affiliations:** 1 Medical Department at Diater Laboratorios, 28919 Madrid, Spain; v.lopez@diater.com; 2 Diater Laboratorios, 28919 Madrid, Spain; r.palacios@diater.com; 3 Pediatric Pulmonology and Allergy Unit, Department of Pediatrics, Dr Peset University Hospital, 46017 Valencia, Spain; tortajadamig@gmail.com; 4 Department of Pediatrics, Obstetrics and Gynecology, University of Valencia, 46010 Valencia, Spain; 5 IVI Foundation, 46026 Valencia, Spain; 6 Research and Development Department at Diater Laboratorios, 28919 Madrid, Spain; 7 Departamento de Parasitología, Facultad de Farmacia, Universidad del País Vasco, 01006 Vitoria, Spain; jorge.martinez@ehu.eus

**Keywords:** *Alternaria alternata*, fungus, fungal allergy, rhinitis, prevalence, Alt a 1, asthma, bioclimatic areas

## Abstract

Prevalence of allergy to fungi is around 3–10%. The most prevalent species involved in sensitizations are *Alternaria alternata, Aspergillus fumigatus, Cladosporium herbarum,* and *Penicillium notatum*. Our main objective was to estimate the prevalence of fungal sensitization and its variation across Spain. Following the ICH-GCP, we recruited 1156 patients from 15 allergy departments in Spain. Hospitals were selected by bioclimatic areas. Patients underwent a skin prick test (SPT) with fungi, pollens, house dust mites, and animal dander. Specific IgE to *Alternaria alternata* and Alt a 1 was assessed in patients with positive SPT to fungi. Of the 233 patients (20.2%) sensitized to at least one of the five fungi tested, 162 (69.5%) were sensitized to *Alternaria alternata* and Alt a 1, of whom 113 (69.8%) were children; 181 (77.7%) were also polysensitized to other allergens. *Alternaria alternata* and Alt a 1 sensitization was present in 25.4% of patients in the Continental area, 12.0% in the Mediterranean area, 7.0% in the Semidesertic area, and 2.3% in the Oceanic area. Prevalence of sensitization to the other tested sources was 63.8% to pollens, 60.5% to house dust mite, and 38.1% to animal dander. We concluded that the prevalence of fungal allergy is increasing. Fungi are still the fourth source of allergen sensitization. *Alternaria alternata* sensitization is the most prevalent in allergic patients to fungi. Alt a 1 is present in almost 90% of the patients sensitized to *Alternaria alternata.*

## 1. Introduction

Allergy to fungi has an estimated prevalence around 3–10% [1,2]. The most prevalent species involved in allergic disease or sensitizations are *Alternaria alternata, Aspergillus fumigatus, Cladosporium herbarum,* and *Penicillium notatum*. *Alternaria alternata* represents 60% of the positive skin prick tests (SPT) among fungi sensitized patients [3].

The European Community Respiratory Health Survey showed that 4.4% of the adult population studied (*n* = 11,355) were sensitized to *Alternaria alternata* [4]. GA2LEN initiative performed a study in 14 countries of the European Community (*n* = 3034) and showed a prevalence of sensitization to *Alternaria alternata* of 9%, with ratios between 2% (Finland) and 23.8% (Greece) [5]. In the United States, the prevalence of positive SPT to *Alternaria alternata* was estimated at around 12.9% in subjects aged between 6 to 74 years old (*n* = 20,322) [6]. In Spain, this prevalence was estimated at around 20% [7].

Fungi are the fourth source of sensitization, after pollens and dust mites, involved in allergic respiratory diseases, *Alternaria alternata* and its major allergen Alt a 1 being the most relevant and most studied in allergic diseases [8,9,10,11,12,13]. Although there are several epidemiologic studies about its prevalence, it varies according to the diagnosis [14,15].

Monosensitization to fungi is rare. This sensitization is usually associated to other allergens [3], and some studies have described the capacity of fungi for activating the immune system and increasing the inflammatory response induced by other allergens such as grass pollen [16]. In addition, fungi sensitization has a strong genetic influence [17] and it is related to an increase of asthma severity and admission to hospitals [18,19,20].

*Alternaria alternata* is considered an outdoor fungus, saprophyte in plants, food, and soil. Its optimal temperature is around 21 °C, but it can survive between 2–32 °C, with humidity conditions greater than 84%. Sporulation takes place between May and November, presenting its higher incidence in summer and autumn [21,22,23]. 

Outdoor spores counting can reach 7500 spores/m^3^ of air; indoor counting is about 280 spores/m^3^ [24]. Indoor presence is associated with high humidity, poor air circulation, and the presence of cockroaches or cat dander, linked to the “sick building syndrome” [25]. A Polish study in adults (*n* = 500) correlated a concentration of 80 spores/m^3^ with a flare-up of allergic symptoms and levels higher than 300 spores/m^3^ are associated with dyspnea [26].

We have estimated the prevalence of *Alternaria alternata* sensitization in Spain and have studied the characteristics of sensitized patients to provide the best diagnosis and treatment.

## 2. Materials and Methods

This is an observational, cross-sectional, and multicentric study aimed to draw a new map of fungal sensitization in Spain, considering bioclimatic areas (areas where the climate, fauna, and flora are similar) [27].

The study was conducted in the allergy department of 15 hospitals located in 6 Spanish regions (Andalusia, Catalonia, Extremadura, Valencian Community, Galicia, and Basque Country). Each department should enroll 100 consecutive patients diagnosed with allergic rhinitis or rhinoconjunctivitis with or without asthma. Patients included were between 3–70 years of age; older than 16 years were considered adults and younger than 16 were considered children.

Participating patients gave informed consent. Demographic variables of the patients were collected (age, sex, previous allergy pathologies, and smoking habits). To classify rhinitis and asthma, the Allergic Rhinitis and its Impact on Asthma (ARIA) guideline [28], the Spanish guidelines for the management of asthma (GEMA 5.0) [29], and the asthma control test (ACT) [30,31] were used. A SPT to fungi, pollens, animal dander, and house dust mites was performed to all patients. SPT was considered positive if the papule diameter was bigger than 3 mm; this SPT were performed with Diater laboratories extracts. Specific IgE to *Alternaria alternata* and to Alt a 1 was assessed in patients who were positive to any of the tested fungi. They were asked to fill a Mold questionnaire (Appendix A and Appendix B).

Primary objective was to describe the sensitization to *Alternaria alternata* and its major allergen Alt a 1 in Spain. To achieve this objective, we calculated the percentage of patients sensitized and its confidence interval at 95% for the whole sample and for each center separately, comparing them with chi-square test.

Secondary objectives were to determine the percentage of patients with positive SPT to the tested fungi, house dust mite, pollens, and animal dander; the percentage of patients with positive IgE to *Alternaria alternata* and Alt a 1 among the patients sensitized to fungi by SPT; and correlate the demographic characteristics of these patients.

After estimating the prevalence of *Alternaria alternata* and Alt a 1 sensitization in each center, patients were grouped by bioclimatic area. The selected areas were Mediterranean, Semidesert, Continental, and Oceanic.

The statistical analysis was carried out by SAS^®®^ v9.3 (SAS Institute Inc., Cary, NC, USA).

## 3. Results

### 3.1. Recruitment

**Figure 1:**
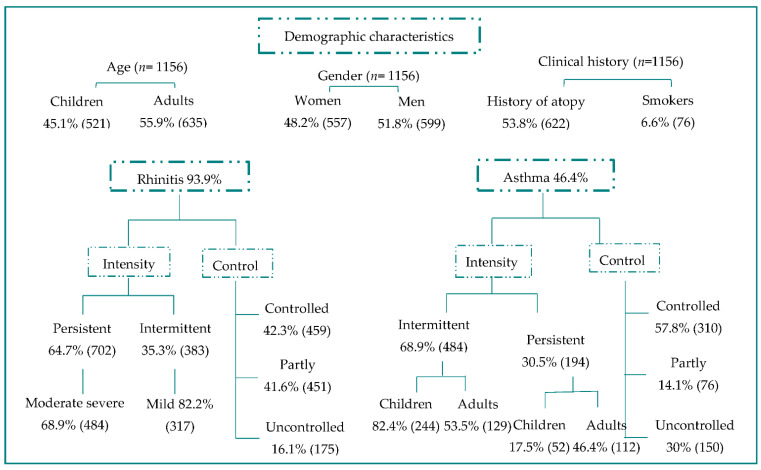
Characteristics of patients included in the study (*n* = 1156).

We recruited a total of 1191 patients, 35 of them were excluded from the analysis per protocol, so we obtained 1156 patients. Patients were excluded because 34 did not have any sensitization and one patient did not want to do the tests. Table 1 shows the distribution of patients by center and Figure 1 shows the characteristics of patients included in the study.

The selected centers were located as follows: Mediterranean region (Barcelona, Manresa, Valencia, Seville, and Huelva); Semidesert region (Almeria); Continental region (Badajoz and Lleida); and Oceanic region (Lugo and Vitoria). 

### 3.2. Primary Objective

The main objective of the study was to determine the percentage of patients sensitized to *Alternaria alternata*/Alt a 1 in each region.

**Figure 2:**
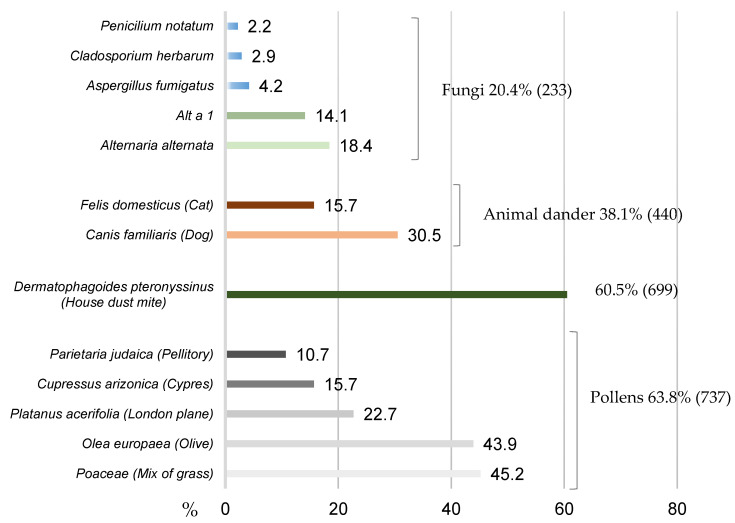
SPT results by allergen source (%).

Figure 2 shows the SPT results by allergen source. Table 2 shows the distribution of patients sensitized to any fungi and *Alternaria alternata* and Alt a 1 by region and Table 3 shows patients sensitized to any fungi and *Alternaria alternata* or Alt a 1 by bioclimatic region.

**Figure 3:**
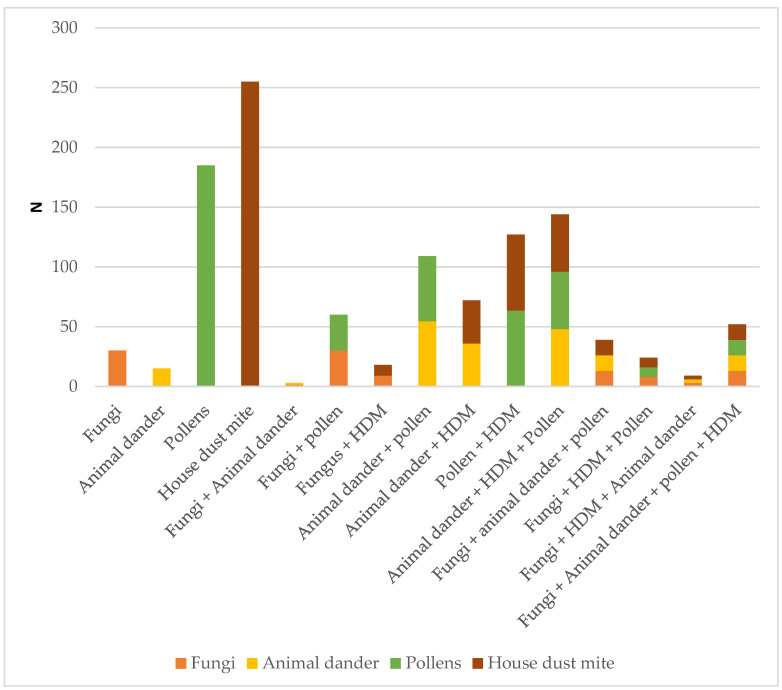
Polysensitization of the study population by allergen groups.

Polysensitization was seen in 56.5% of the study population. Of patients sensitized to any fungi, 77.7% were also polysensitized to other allergens. Figure 3 shows the distribution of patients by allergen groups.

Among the patients sensitized to any of the fungi tested (233); 214 patients had allergic rhinitis; description of these patients is below in Table 4.

### 3.3. Secondary Objectives

**Figure 4:**
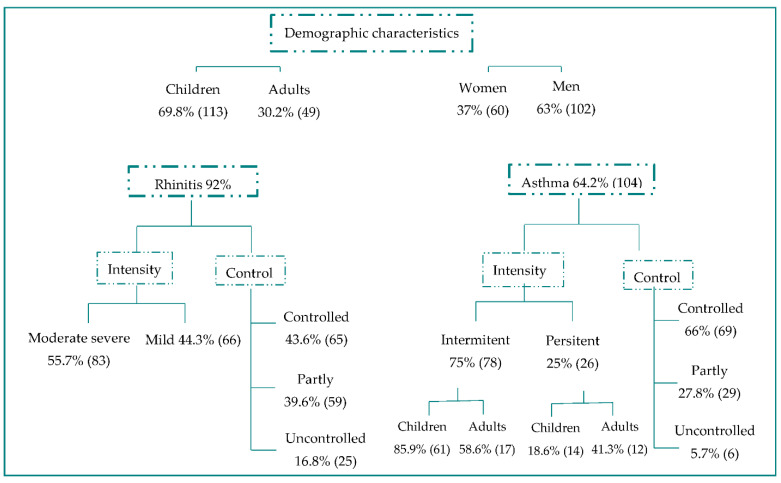
Characteristics of patients sensitized to Alternaria alternata and Alt a 1.

We analyzed different variables among the sensitized subjects to *Alternaria alternata* and Alt a 1. Figure 4 shows the characteristics of patients sensitized to *Alternaria alternata* and Alt a 1.

In total, 113 (69.8%) of the children were sensitized to *Alternaria alternata* and Alt a 1 and 49 (30.2%) of adults. There was a significant (*p* = 0.0387) difference between the percentage of children sensitized to Alternaria alternata and that of those non-sensitized.

In total, 102 (63%) of patients sensitized to *Alternaria alternata* and Alt a 1 were men; a significant difference (*p* = 0.0292) (2:1 male: female) was found between those sensitized to *Alternaria alternata* and those who were not.

There have been no significant differences found between patients sensitized to *Alternaria alternata* and Alt a 1 and those who were not sensitized regarding asthma and rhinitis or its control. 

Positive SPT to *Alternaria alternata* was associated to the patients who had specific IgE to *Alternaria alternata* and Alt a 1: 167 (88.8%) of the patients with positive SPT to *Alternaria alternata* had specific IgE to *Alternaria alternata* and Alt a 1, and 134 (91.2%) with positive SPT to Alt a1 had specific IgE to *Alternaria alternata* and Alt a 1.

## 4. Discussion

This study estimates the prevalence of sensitization to fungi in Spain, focusing on *Alternaria alternata*.

Our results showed that fungi are the fourth cause of allergic sensitization, after pollens and dust mites; in line with *Alergológica* [32] and D’Amato et al. [7]. We have observed that 233 (20.2%) of patients were sensitized to at least one of the five fungi tested. Among these 233 patients, 219 (94.0%) were sensitized to *Alternaria alternata* and/or Alt a 1, and 162 (69.5%) were sensitized to both *Alternaria alternata* and Alt a 1, which represents 14% of the sample accordingly with the 10–13% reported in 2015 [32].

Prevalence of sensitization to *Alternaria alternata* was higher in Extremadura and Catalonia followed by Andalusia and Basque Country; lower sensitization was found in Valencian Community and Galicia. Compared to the prevalence of sensitization in patients with rhinitis studied in 2015 in Spain, we have seen that the prevalence in general has increased and we perceived differences in some regions.

In five of the six regions studied, the sensitization to fungi doubled its prevalence. On the other hand, Galicia has shown a decrease from 6.5% to 1%. This decrease might be due to the population recruited. Most of patients were adults and the consecutive recruitment could have influenced.

In general, we have detected a higher prevalence of sensitization to *Alternaria alternata* in the inner regions of Spain, such as Lleida with a prevalence of 45% and Badajoz with 21.5%.

The good correlation between SPT and serologic test (specific IgE) for *Alternaria alternata* and Alt a 1 is noteworthy. Thus, Alt a 1 should be one of the allergens tested in the SPT regular allergen battery, because this protein gives us precise information about patient sensitization and helps us in prescribing the best treatment option for each patient.

Most patients sensitized to any fungi (203, 87.1%) were polysensitized to other allergen sources, mainly pollens, such as olive and grass [33]. At this point we would like to underline the perception of *Alternaria alternata* being a trigger allergen to the atopic march, due to its high prevalence in children and the high rate of polysensitization when they grow up, and the importance of an early treatment in order to prevent further sensitizations. Does this sensitization decrease as we get older and become sensitized to other sources?

Cross reactivity among fungi is not well studied but there are some statements about it. From a phylogenetical point of view, there are two classes of cross reactivity: species-specific and cross-reactive allergens. Among them, Asp f 1, Alt a 1, Cop c 1, and Mala s 1 represent specie-specific proteins that cannot be found in any other genera. However, except these and perhaps some other few exceptions, most of the fungal allergens represent cross-reactive structures covering different protein families [34]. In this study we saw that most of our patients were sensitized to *Alternaria alternata* or Alt a 1, which could explain that the most prevalent fungal genera among allergic patients in Spain is *Alternaria alternata.* As we see (Table 4) this sensitization to fungus relays on allergic pathology and we can consider all of them, most of all due to its prevalence *Alternaria alternata*, a relevant cause of sensitization and allergens to take into account when diagnosing patients. 

Regarding our studied population, almost 70% of patients sensitized to *Alternaria alternata* were younger than 16 years old, even though there were more adults included in our studied population and 63% were male. Children sensitized to both allergens (*Alternaria alternata* and Alt a 1) had a worse control of allergic pathology.

Patients sensitized to *Alternaria alternata* have moderate to severe partly controlled allergic rhinitis according to ARIA, and episodic asthma partly controlled according to GEMA 5.0. [29] This sensitization is statically related to asthma. *Alternaria alternata* sensitization is supposed to be a risk factor for developing asthma.

Chew’s questionnaire [35], which is based on identifying a relation among house and environment [36,37] characteristics and presence of fungi, shows that patients’ sensitization worsens at the end of summer and autumn. They do not seem to be affected by other items such as rain, humidity, or fungi at their home.

## 5. Conclusions

We concluded that fungi are an important source of sensitization in Spain and its incidence is increasing; patients have mild control of their symptoms in general and they might have benefits from an early diagnosis and treatment in order to prevent them from developing asthma and other sensitizations.

Our unmet needs and proposal of new studies are to further study the relationship between sensitization and atopic march, and to relate polysensitization to environment conditions and exposures.

We have updated the Spanish fungal map relating it to regions.

## Figures and Tables

**Table 1:** Distribution of patients included in the study by center.

Centers	Children	Adults	Total (%)
Andalusia	126	269	395 (34.1)
Catalonia	181	106	287 (24.8)
Extremadura	96	99	195 (16.9)
Valencian Community	98	50	148 (12.8)
Galicia	18	82	100 (8.7)
Basque Country	2	29	31 (2.7)
Total	521	635	1156 (100)

**Table 2:** Patients sensitized to fungi and *Alternaria alternata* and Alt a 1 by center.

Centers	Patients Included N	Positive SPT to Any Fungi N (%)	Positive IgE to *A. alternata* and Alt a 1 N (%)	Positive SPT to *A. alternata* and Alt a 1	
				Children N (%)	Adults N (%)
Andalusia	395	78 (19.7)	55 (13.9)	20 (5.1)	28 (7.1%)
Catalonia	287	81 (28.2)	51 (17.8)	34 (11.8)	22 (7.7%)
Extremadura	195	53 (27.2)	41 (21.0)	30 (15.4)	12 (6.2%)
Valencian Community	148	15 (10.1)	12 (8.1)	8 (5.4)	2 (1.4%)
Galicia	100	1 (1.0)	1 (1.0)	1 (1.0)	0 (0%)
Basque Country	31	5 (16.1)	2 (6.5)	0 (0)	2 (6.5%)
Total	1156	233 (20.2)	162 (14.0)	93 (8.0)	64 (5.5%)

**Table 3:** Patients sensitized to fungi and *Alternaria alternata* or Alt a 1 by bioclimatic regions.

Bioclimatic Areas	Patients Included N	Positive SPT to Any Fungi N (%)	Positive IgE to *A. alternata* or Alt a 1 N (%)
Mediterranean	592	124 (20.9)	71 (12.0)
Semidesert	201	27 (13.4)	14 (7.0)
Continental	232	76 (32.8)	59 (25.4)
Oceanic	131	6 (4.6)	3 (2.3)
Total	1156	233 (20.2)	147 (12.7)

**Table 4:** Presence (%) of allergic rhinitis in sensitized patients to each fungal allergen.

Fungus	Sensitized (*n* = 233)	With Allergic Rhinitis (*n* = 214)/%
*Alternaria alternata* and Alt a 1	162	149/92%
*Cladosporium herbarum*	31	31/100%
*Aspergillus fumigatus*	45	43/95.5%
*Penicilium notatum*	24	23/95.8%

## Data Availability

Authors ensure that data shared are in accordance with consent provided by participants on the use of confidential data, following *Reglamento (UE) 2016/679 del Parlamento europeo y del Consejo de 27 de abril de 2016 de Protección de Datos (RGPD)* and the good clinical practice and declaration of Helsinki. The data presented in this study are available on request from the corresponding author. The data are not publicly available due to GDPR.

## Appendix A. Mold Questionnaire Part 1

**Sensitization**	***Alternaria alternata*** **/Alt a 1**	
**Part 1**	**N**	**%**
1. Do your symptoms worsen when you are at a place with fungi or dump patches?	161	100.0
▪ Yes	76	47.2
▪ No	85	52.8
2. Do your symptoms worsen when you are at an old building?	162	100.0
▪ Yes	76	46.9
▪ No	86	53.1
3. Do your symptoms worsen when you go to the basement?	160	100.0
▪ Yes	52	32.5
▪ No	108	67.5
4. Do your symptoms worsen when you cut the grass or move leaves or compost?	160	100.0
▪ Yes	71	44.4
▪ No	89	55.6
5. Do your symptoms worsen after a storm?	161	100.0
▪ Yes	50	31.1
▪ No	111	68.9
6. Do your symptoms worsen when you are at a building with fungi odors or dump patches?	161	100.0
▪ Yes	81	50.3
▪ No	80	49.7
7. Do your symptoms worsen at the end of summer/autumn?	161	100.0
▪ Yes	97	60.2
▪ No	64	39.8

## Appendix B. Mold Questionnaire Part 2

**Sensitization**	***Alternaria alternata*** **/Alt a 1**	
**Part 2**	**N**	**%**
1. Have you had at your house humidity problems, water filtrations or floods in the last 12 months? Have you had done any restoration work due to humidities or water damages?	162	100.0
▪ Yes	37	22.8
▪ No	125	77.2
2. In the past 12 months, how often have you had noticed odors or dump patches at your home?	162	100.0
▪ Diary	9	5.6
▪ Weekly	4	2.5
▪ Monthly	4	2.5
▪ Few times a year	37	22.8
▪ Never	108	66.7
3. Have you observed in the past 12 months dump patches bigger than 20 × 30 cm at your home?	162	100.0
▪ Yes	42	25.9
▪ No	120	74.1
4. Have you observed in the past 12 months condensations at the windows?	162	100.0
▪ Yes	55	34.0
▪ No	107	66.0
5. Have your furniture, clothes or any of your possessions suffered water damages?	162	100.0
▪ Yes	7	4.3
▪ No	154	95.7
